# Advanced aging phenotype is revealed by epigenetic modifications in rat liver after *in utero* malnutrition

**DOI:** 10.1111/acel.12505

**Published:** 2016-07-29

**Authors:** Hye J. Heo, Jessica N. Tozour, Fabien Delahaye, Yongmei Zhao, Lingguang Cui, Nir Barzilai, Francine Hughes Einstein

**Affiliations:** ^1^ Department of Obstetrics & Gynecology and Women's HealthAlbert Einstein College of Medicine1300 Morris Park AveBronxNY10461USA; ^2^ Department of GeneticsAlbert Einstein College of Medicine1300 Morris Park AveBronxNY10461USA; ^3^ Department of MedicineAlbert Einstein College of Medicine1300 Morris Park AveBronxNY10461USA

**Keywords:** aging, DNA methylation, liver, maternal overnutrition, maternal undernutrition

## Abstract

Adverse environmental exposures of mothers during fetal period predispose offspring to a range of age‐related diseases earlier in life. Here, we set to determine whether a deregulated epigenetic pattern is similar in young animals whose mothers’ nutrition was modulated during fetal growth to that acquired during normal aging in animals. Using a rodent model of maternal undernutrition (UN) or overnutrition (ON), we examined cytosine methylation profiles of liver from young female offspring and compared them to age‐matched young controls and aged (20‐month‐old) animals. HELP‐tagging, a genomewide restriction enzyme and sequencing assay demonstrates that fetal exposure to two different maternal diets is associated with nonrandom dysregulation of methylation levels with profiles similar to those seen in normal aging animals and occur in regions mapped to genes relevant to metabolic diseases and aging. Functional consequences were assessed by gene expression at 9 weeks old with more significant changes at 6 months of age. Early developmental exposures to unfavorable maternal diets result in altered methylation profiles and transcriptional dysregulation in *Prkcb, Pc, Ncor2,* and *Smad3* that is also seen with normal aging. These Notch pathway and lipogenesis genes may be useful for prediction of later susceptibility to chronic disease.

## Introduction

David Barker was one of the first to suggest that adaptive responses to adverse *in utero* environment could have a negative impact on future functional capacity (Barker & Osmond, 1986). The observation of poor early developmental nutrition leading to long‐term metabolic impartment has lead to the evolution of the ‘thrifty phenotype’ hypothesis, which specifies how substandard environment during fetal development can affect tissue metabolic capacity and thus influence disease risk (Hales & Barker, 2013). While the validity of this hypothesis has been well accepted, the underlying mechanism linking the environmental exposure to genetic outcome has not been fully elucidated. The myriad of intrauterine conditions leading to low birthweight or intrauterine growth restriction show increased risk for obesity, type 2 diabetes, hypertension, and cardiovascular heart disease in adulthood (Barker & Osmond, 1986; Barker *et al*., 1989; Ravelli *et al*., 1998). Interestingly, at the other extreme of fetal growth, infants born large for gestational age, used as an indicator of exposure to excess nutrients, are also at greater risk for these chronic age‐related diseases in addition to some cancers (Lahmann *et al*., 2004; Dyer & Rosenfeld, 2011).

Although an individual's phenotypic age and lifespan is strongly influenced by genetic makeup, the manifestation of age‐related diseases results from an intricate interplay between both genetic and environmental factors. There is evidence that these factors may strengthen DNA methylation stability or influence its deviation between genetically identical individuals over time (Shah *et al*., 2014). Studies of monozygotic twins demonstrate widening epigenetic divergence over time, which correlates to greater interindividual variability in phenotype (Fraga *et al*., 2005). Fraga *et al*. (2005) showed growing DNA methylation differences with age, especially in twin pairs whose lifestyle, location, and/or health status were disparate (Fraga *et al*., 2005), defining DNA methylation as a dynamic mark able to record a variety of exposures at the cellular level. Recent studies have also demonstrated that methylation across tissues and cell types can be correlated with chronological age, indicating that DNA methylation certainly works as a function of age or can be accelerated by different conditions, most notably in the setting of cancer (Hannum *et al*., 2013).

DNA methylation and histone modifications are both heritable and responsive to environmental cues. DNA methylation is considered to be the most stable of epigenomic regulators (Byun *et al*., 2012) that has been used to identify alterations associated with different environmental insults and normal aging and age‐related disease. For instance, our group has shown a global DNA hypomethylation profile in rat liver associated with normal aging. These alterations were targeted toward metabolically relevant loci and correlate with alterations in gene expression (Thompson *et al*., 2010b). A similar profile of global DNA hypomethylation associated with age is seen in human cells (Fuke *et al*., 2004). The relationship of altered DNA methylation and specific age‐related diseases, such as type 2 diabetes, has been demonstrated using a candidate gene approach. Increased methylation in the distal promoter and enhancer regions of the pancreatic duodenal homeobox 1 (*PDX1*), a key pancreatic developmental transcription factor, is seen in association with diabetes (Yang *et al*., 2012). More recently, a positive correlation was found between high body mass index and advanced DNA methylation age of human liver (Horvath *et al*., 2014). These studies suggest that over the lifetime of an individual, serial exposures and the resulting accumulated epigenomic effects may ultimately lead to functional impairment and clinical disease.

Fetal life is a critical period of developmental plasticity during which insults can give rise to distinctive morphological states (Bateson *et al*., 2004). While the environment over the course of a lifetime can have a cumulative impact on epigenetic dysregulation, adverse exposures in early life may set alterations in epigenetic markings within young replicative cells. Targeting replicative cells holds greater potential to propagate those epigenetic changes into later life, thereby acting as an epigenetic memory of early life conditions. The effect of adverse maternal diet on locus‐specific differential methylation has been demonstrated in liver (Dudley *et al*., 2011; Xie *et al*., 2015), but a genomewide approach specific to liver is limited. However, our group interrogated genomewide methylation changes associated with intrauterine growth restriction in the pancreatic islets of male rat offspring and found methylation changes occurring in intergenic regions of pathways regulating β‐cell function (Thompson *et al*., 2010a). We also demonstrated that DNA methylation profiles are similar in growth restricted and over‐grown infants, but distinct from control subjects in CD34^+^  hematopoietic stem and progenitor cells (Delahaye *et al*., 2014). In regard to these findings, we implemented our genomewide analytical approach on the liver, a highly metabolic and actively replicating tissue. Due to its role in lipid and glucose homeostasis and interactions with adipose, muscle, and nervous system, it has clear implications for aging and metabolic health (Bechmann *et al*., 2012).

Therefore, we examined genomewide DNA methylation in the liver of animals exposed to maternal malnutrition. The use of an animal model allows for greater control of exposure conditions and decreasing variability related to diet and activity in human studies. Furthermore, we incorporate samples at different ages under normal conditions or after exposure providing a multitime point investigation as well as comparison studies in naturally aged animals. These unique analyses led to the identification of pathways targeted for dysregulation that may be indicative of an advanced aging phenotype associated with adverse *in utero* nutrition mediated by a cellular memory.

## Results

### Established animal models of maternal under‐ and overnutrition

**Figure 1:**
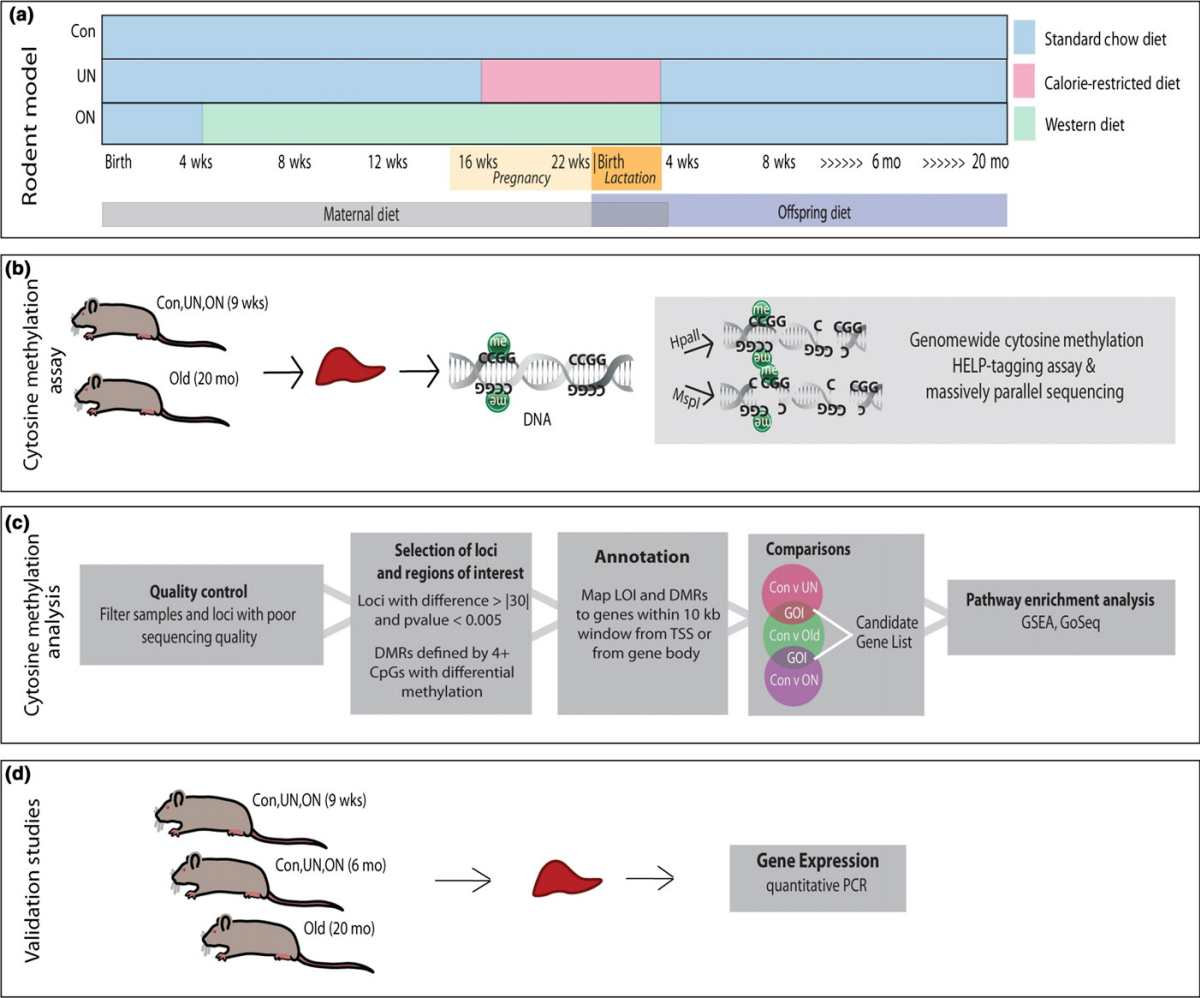
Summary of Experimental Protocol. Four groups of rats were established for dna methylation studies: (1) 9‐week‐old offspring of control (Con) dams on standard diet *ad libitum*; (2) 9‐week‐old offspring of maternal undernutrition (UN) dams exposed to 50% of standard chow consumed by controls starting on gestational day 11 and continued through lactation; (3) 9‐week‐old offspring of maternal overnutrition (ON) dams started on western diet *ad libitum* starting 3 weeks of age and continued on the diet during pregnancy and lactation; (4) aged (Old) rats maintained on standard chow from weaning to 20 months of age (a). Genomewide cytosine methylation was interrogated using HELP‐tagging assay which measures differential enzymatic digestion based on the presence of methylation, MspI (methylation‐insensitive) and HpaII (methylation‐sensitive) (b). Significant differential methylation of single loci (LOI) and/or regions (DMRs) of interest was mapped to genes within 10 kb of TSS or within gene body. Gene set enrichment analysis (GSEA) and GoSeq pathway enrichment analysis led to the identification of genes (GOI) and pathways of interest (c). Biological validation performed by quantitative PCR (d).

We utilized established models of maternal undernutrition (UN) (Torrens *et al*., 2014) and maternal overnutrition (ON) (Sen & Simmons, 2010) feeding, during pregnancy and lactation in Sprague‐Dawley rats. In the undernutrition model, offspring birthweight is decreased compared to offspring of control dams, while offspring of overnutrition dams show little to no difference (Sen & Simmons, 2010). However, as these animals age both under‐ and overnutrition, exposed offspring have increased weight and BMI relative to controls (Desai *et al*., 2015). These models also result in metabolic abnormalities and decreased insulin sensitivity in adulthood. Figure 1a summarizes the dietary schedule for each study group. Both control (Con) animals, fed standard chow, and ON dams are fed their distinct diets *ad libitum*. Notably, the overnutrition model mimics a western diet that is not a model of excess caloric intake, but rather one of isocaloric malnutrition, consisting of higher percent fat and saturated fats (custom made by Harlan Tekland‐see Experimental methods). To control for litter size, litters were culled to 8 on the first day of life. All animals were weaned at the end of 3 weeks of age and placed on standard chow diet *ad libitum* until 9 weeks of age, the time of epigenetic profiling, or 6 months of age, for gene expression analysis. Old animals were aged to ~20 months on standard chow from weaning.

### Distinct patterns of DNA methylation in rats exposed to adverse developmental exposure mimics aging

A genomewide cytosine methylation assay was performed on liver tissue isolated from Sprague‐Dawley rats in each of four study groups: young 9‐week‐old control (Con,*n* = 5), undernutrition (UN, *n* = 6) and overnutrition (ON, *n* = 5) and unexposed 20 month old (Old, *n* = 6). The HELP‐tagging assay used interrogates methylation of ~1.6 million loci in the rat genome. Following quality control measures (see Experimental methods), 617998, 709778, 710027 loci remained from Con vs. ON, Con vs. UN, and Con vs. Old, respectively, and 614,084 loci between all three comparisons were further analyzed utilizing a validated pipeline. (Fig. 1b–d) (Delahaye *et al*., 2014).

**Figure 2:**
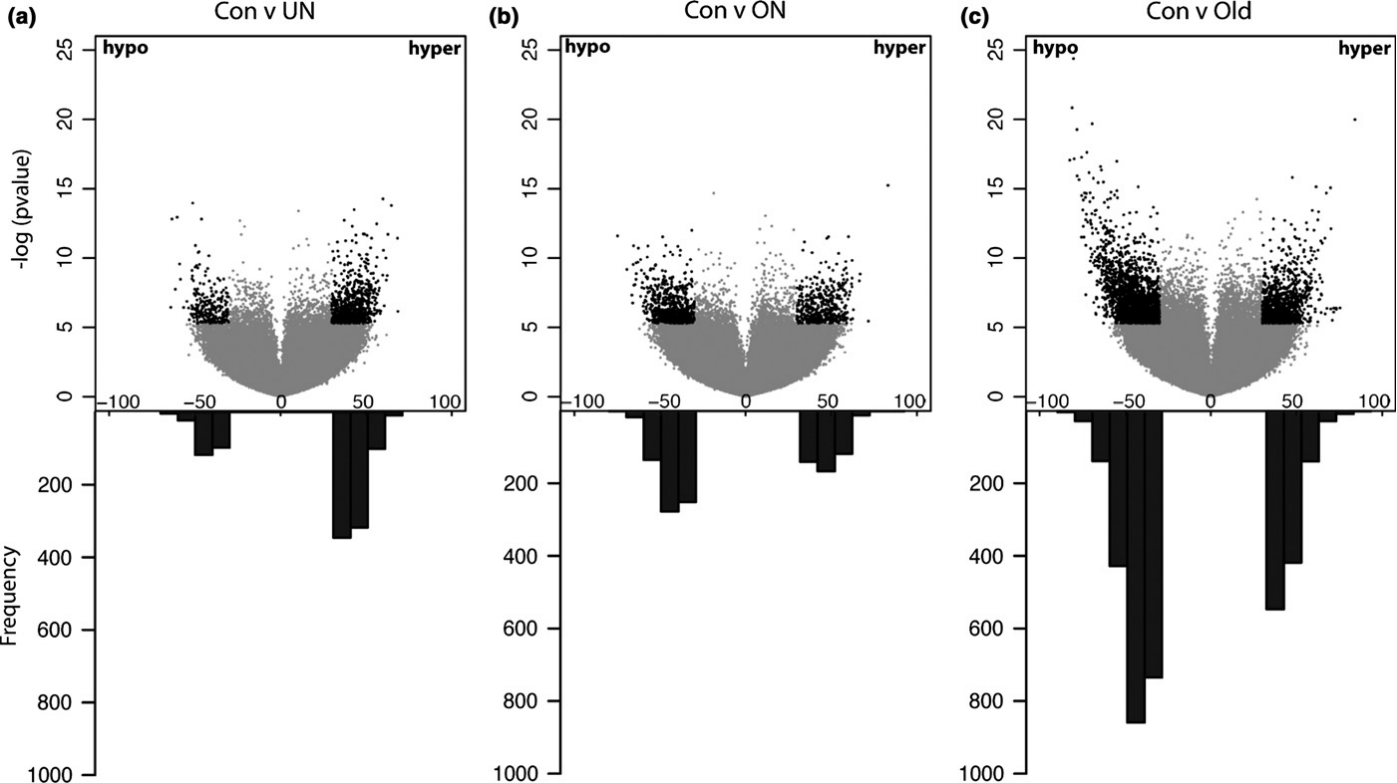
Genomewide methylation analysis. Volcano plots illustrating the distribution of hypo‐ and hypermethylated loci in 9‐week UN (a), 9‐week ON (b), 20‐month Old offspring (c) compared to 9‐week controls with histogram illustrating the frequency of loci of interest (LOI). LOI are determined by a threshold of > absolute methylation difference of 30 and significance threshold of < *P*‐value 0.005 (black dots).

Global patterns of methylation were first evaluated in Con with comparison to UN, ON, and subsequently Old. Unsupervised clustering of the 614,084 loci from all four groups demonstrates no global shifts in methylation patterns among all groups, due to lack of segregation of groups (Fig. S1a). Whereas exposure to maternal overnutrition does not result in an overall shift toward hypo‐ or hypermethylation (Fig. 2a,b), undernutrition is associated with hypermethylation (Fisher's exact test, *P* < 0.001) and aging with an enrichment of more hypomethylated loci (Fisher's exact test, *P* < 0.001) in the liver (Fig. 2c), as previously seen (Thompson *et al*., 2010b).

**Figure 3:**
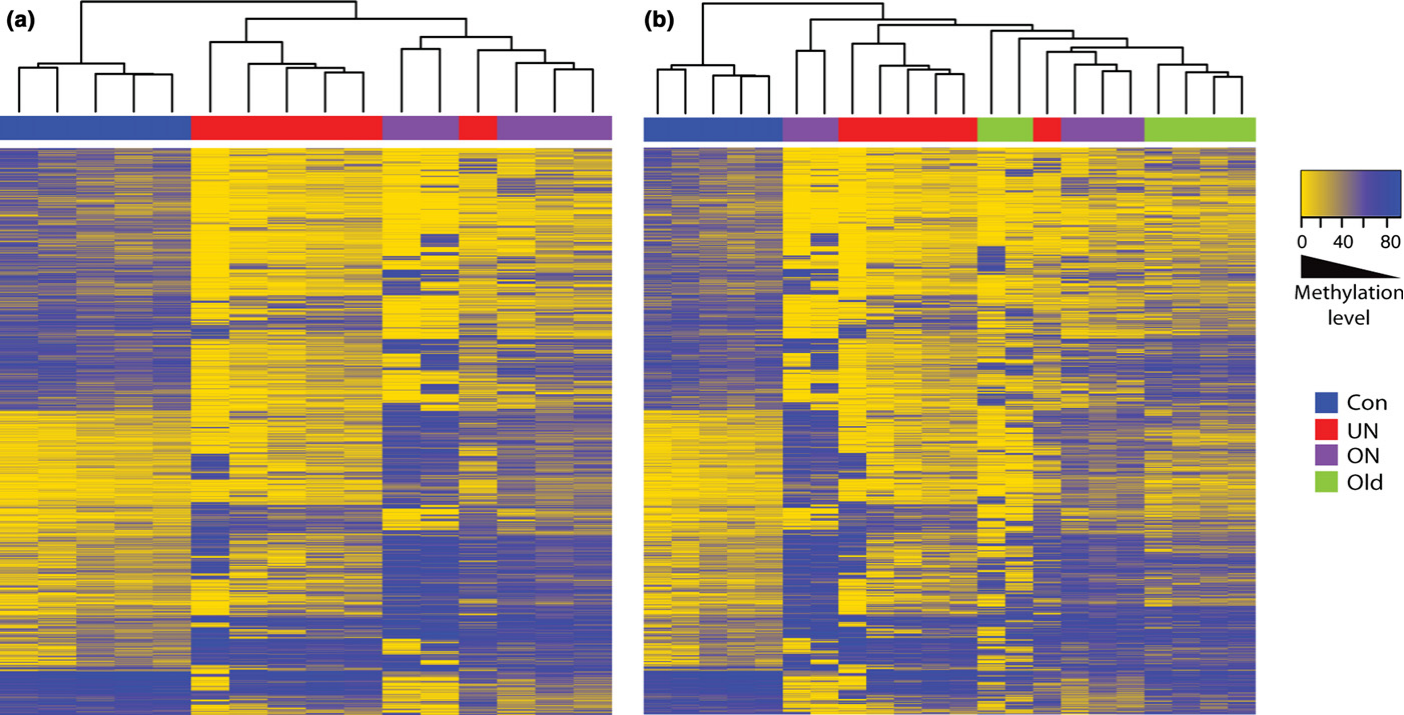
Comparison of methylation profiles. Clustering analysis of the 1906 loci combined from the LOI in Con v UN and Con v ON (a). Clustering analysis including methylation profile in Old for the same 1906 loci (b).

Candidate loci were identified with significant changes in methylation defined as an absolute difference in mean methylation scores >30% and a *P‐* value <0.005 (*t*‐test). This cutoff was determined to allow a detection power >80%. A self‐organizing heat map demonstrates the top 1,906 differentially methylated loci combined from UN vs. Con and ON vs. Con. A distinct pattern of methylation is shared between UN and ON compared to Con (Fig. 3a). Furthermore, clustering analysis indicates that the methylation profiles of these loci in the exposed 9‐week groups are more similar to Old animals than to their age‐matched controls (Fig. 3b). Young offspring exposed to adverse maternal diets demonstrate methylation patterns mimicking that of old animals. This pattern emphasizes the vulnerability of select loci, as we do not see this represented when taking 1906 random loci under unsupervised clustering (Fig. S1b). To further narrow the list of candidate loci, we focused on those loci involved in both exposure and aging. We identified 98 overlapping significantly differentially methylated loci between UN vs. Con and Old vs. Con, 133 loci between ON vs. Con and Old vs. Con, and 24 loci in all three comparisons (Fig. S2a).

### Association of DNA methylation changes to genes relevant to metabolism and aging

Differentially methylated loci were mapped to RefSeq genes if they were located within a window from 10‐Kb upstream of the transcription start site and 10‐kb downstream of the gene body. The criteria used to map loci to genes were designed to capture putative gene regulatory sites (Everett *et al*., 2013). This identified 168 genes correlating with differential methylation in both UN and Old compared to Con, 220 genes in both ON and Old compared to Con, and 40 genes in all three comparisons (Fig. S2b).

As a secondary approach, differentially methylated regions (DMRs) were identified to increase the likelihood of identification of candidate genes with biological significance. DMRs were defined as having four or more consecutive differentially methylated loci within a 10‐kb distance of each other. We adapted the use of ‘Bump‐hunting’ using the *dmrFind* function through ‘charm’ package (Aryee *et al*., 2011). DMRs were mapped using similar window approach to 85 genes in UN, 210 genes in ON, and 89 genes in Old compared to Con. A representative DMR in *Cacna1d* gene is illustrated in Fig. S3. Genes identified through both single locus and DMR analyses were prioritized within the candidate loci.

To determine pathways that may be preferentially targeted for DNA methylation dysregulation, candidate genes were uploaded to gene set enrichment analysis (GSEA) (Subramanian *et al*., 2005) and analyzed with the Kyoto Encyclopedia of Genes and Genomes (KEGG) pathway database. The ‘GoSeq’ package (Young *et al*., 2010) was utilized to normalize the genes based on number of HpaII sites per gene to eliminate bias. Pathways significantly represented from the GoSeq analysis are summarized in Table 1. *Notch* and *Calcium signaling* pathways were significantly enriched in all comparisons. Con v UN v Old comparison demonstrates enrichment in p53 signaling, cancer, and cell cycle pathways known to be involved in aging and disease progression. Con v ON v Old comparison identified enrichment of cardiovascular‐focused pathways. These results suggest both common and distinct mechanisms for development of age‐related disease.

**Table 1:** KEGG pathways from each set of comparisons to old

KEGG Pathways	Genes in Pathway	Differentially Methylated Genes	Significance of Enrichment (*P*‐value)
Con v UN v Old			
Ca^+2^ signaling	169	11	0.004
Pancreatic secretion	96	7	0.006
Notch signaling	40	3	0.017
P53 signaling	61	3	0.019
Long‐term potentiation	63	6	0.027
Cancer	283	10	0.030
Chronic Myeloid Leukemia	68	4	0.031
Cell cycle	113	4	0.032
Extracellular matrix receptor	60	4	0.037
NK‐cell‐mediated cytotoxicity	90	4	0.041
Gastric acid secretion	70	5	0.042
Small cell lung cancer	69	4	0.045
Con v ON v Old			
Vascular smooth muscle contraction	107	10	0.004
Notch signaling	40	4	0.005
Mucin type O‐Glycan biosynthesis	25	4	0.020
Dilated cardiomyopathy	78	7	0.023
Ca^+2^ signaling	169	11	0.036
Con v UN v ON v Old			
Notch signaling	40	2	0.007
Fc‐gamma receptor‐mediated phagocytosis	84	3	0.011
Dilated cardiomyopathy	78	3	0.016
Long‐term potentiation	63	3	0.018
GnRH signaling	91	3	0.022
Ca^+2^ signaling	169	4	0.027
Chemokine signaling	168	3	0.029
Chronic Myeloid Leukemia	68	2	0.033
Bile secretion	70	2	0.034
Vascular smooth muscle contraction	107	3	0.043
Progesterone‐mediated oocyte maturation	81	2	0.045

### Gene expression analysis

**Figure 4:**
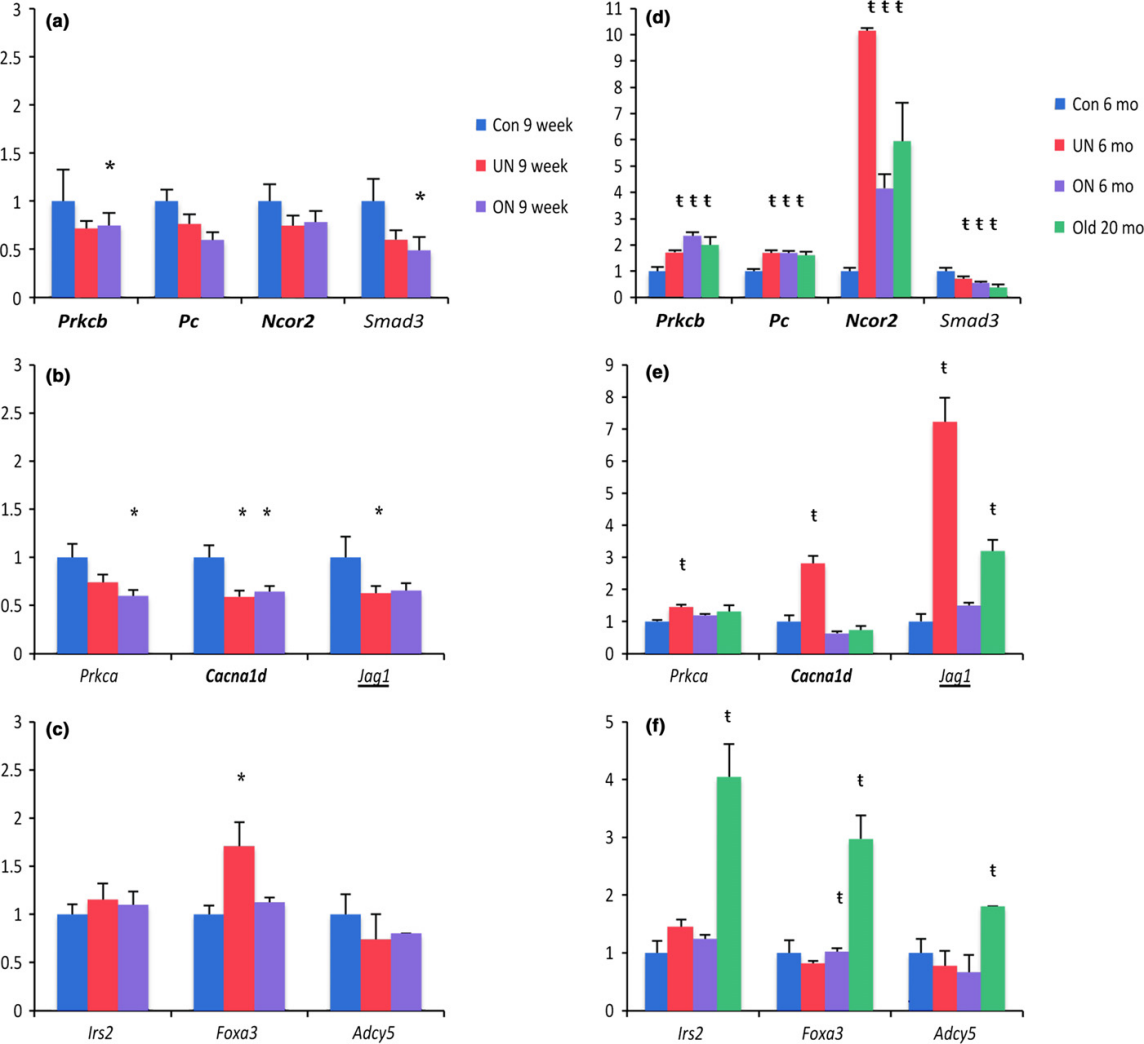
Candidate gene expression studies. Relative fold change calculated by 2^−ΔΔCt^ method, showing mean +/− standard error of the mean (SEM). Fold change normalized to 9‐week controls (a,b,c) or 6‐month controls (d,e,f). Significance calculated by ANOVA followed by Tukey's HSD test; (*) *P*‐value < 0.05 compared to 9‐week Con and (ŧ) *P*‐value < 0.05 compared to 6‐month Con. Genes with DMRs called in UN 9 week in bold, genes with DMRs called in Old are underlined.

Next, gene expression of candidate genes was examined to determine whether observed DNA methylation changes identified regions with immediate or long‐term functional alterations. To combine relevance and feasibility, our approach was limited to genes selected to be part of metabolic disease and aging relevant pathways such as Notch, pancreatic secretion, p53, cell cycle, and chemokine signaling. Briefly, a gene was selected (i) if associated to differentially methylated loci in at least two of the three comparisons (Con/UN, Con/ON, Con/Old) and/or had a differentially methylated region, and (ii) found in one of the above pathways using gene set enrichment analysis. Regarding their short‐term effect, among the 10 tested genes, only four and three genes demonstrated significant expression change in overnutrition and undernutrition animals, respectively, when compared to control at 9 weeks old (Fig. 4a–c). These findings are consistent with developmental programming, in that functional changes related to disease processes may not be apparent early in life but are later unmasked throughout the lifespan. To determine whether the selected candidate genes are identifying genomic regions with long‐term vulnerability, we further evaluated the expression of these genes in 6‐month animals and compared them with control 20‐month‐old animals. We found a substantial effect on expression at 6 months rather than 9 weeks when looking at control versus exposed groups. When comparing to Old control animals, we were able to identify different profiles of expression. First, 6‐month under and overnutrition offspring exhibit profiles similar to Old animals with increased expression of *Prkcb, Pc,* and *Ncor2* and decreased expression of *Smad3* (Fig. 4d). *Ncor2* shows the greatest upregulation of transcription with 10‐, fourfold, and sixfold increase in UN and ON at 6 months, and Old, respectively, compared to 6‐month‐old Con; second, genes with specific pattern of expression for 6 month old UN (*Prkca, Cacna1d,* and *Jag1*, Fig. 4e); and third, genes specific to aging not yet alterated in exposed animals (*Irs2, Foxa3,* and *Adcy5,* Fig. 4f). These genes may be more resistant to perturbation, may not become dysregulated until disease has been established, or may require more time to acquire additional aberrant regulation. Interestingly, the expression profiles found in Fig. 4d,e illustrate that exposure to maternal adverse diet during development increases the susceptibility to chronic disease and mimics an aging gene expression profile.

## Discussion

In this study, we detected early liver methylation alterations using a genomewide approach in a well‐phenotyped animal model of fetal exposure to two extremes in maternal nutrition. Modifications to DNA methylation were further linked to functional consequences later in life. DNA methylation, due to the stability and heritability to daughter cells, is an ideal candidate for a lifelong marker of exposure (Chong & Whitelaw, 2004). Differential DNA methylation in gene regulatory sites alters gene transcription establishing functional relevance (Wijetunga *et al*., 2014). We demonstrate that exposure to adverse maternal nutritional environments leads to acquisition of epigenome markers in vulnerable regions that provide a cellular memory and impart a risk for age‐related disease. Moreover, we show that two extremes in maternal nutrition, undernutrition, and overnutrition exhibit similar epigenetic and expression changes in genes that may converge on analogous disease development.

The liver is a key player in metabolic homeostasis, responsible for gluconeogenesis, glycogen homeostasis, and lipid metabolism. Aging and age‐related diseases are complex and multifaceted, often resulting in a decline or change in function of differentiated cells, a depletion or lack of mobilization of stem cell pools, as well as a shift in cell composition within tissues (Liu & Rando, 2011; Duscher *et al*., 2014). Our integrative analysis allowed us to identify altered loci in exposed animals from key pathways such as Notch, fatty acid, and glycogen metabolism that may play significant roles in mediating this accelerated aging phenotype.

We found differential methylation and expression changes in, *Ncor2*,* Jag1*,* Smad3*, implicating the Notch signaling pathway in our 6‐month‐old UN and ON exposed and unexposed aging animals. The Notch pathway is highly conserved and primarily involved in cell differentiation and tissue development (Kao *et al*., 1998). In the liver, Notch signaling is essential in the development of the biliary system as well as regulating transcription factors responsible for hepatoblast differentiation (Valenti *et al*., 2013). Dysregulation of the Notch pathway has been correlated with increases in hyperglycemia, insulin resistance, and adipose deposition in the liver (Valenti *et al*., 2013). Nuclear receptor corepressor 2 (NCOR2) is an important regulator of hormone receptor target genes through its interactions with histone deacetylases. Complexes containing NCOR2 and HDAC1 have a transcriptionally repressive influence at Notch target sites (Kao *et al*., 1998). Aging is associated with increased expression of *Ncor2* in major metabolic tissues, including liver, adipose tissue, and skeletal muscle. In addition, genetic manipulation shifting NCOR2 binding affinity to its RID2 domain results in premature aging with reduction of mitochondrial function and antioxidant gene expression (Reilly *et al*., 2010). NCOR2 may be a potential therapeutic target with maternal high‐fat diet linked to decreased antioxidant enzyme gene expression in offspring with protection through maternal administration of antioxidants (Zhang *et al*., 2011). Notch dysregulation via JAG1, a well‐conserved activating ligand of the Notch receptor (Golson *et al*., 2009), was demonstrated in the unexposed aging animals compared to 6‐month‐old controls, with an exacerbated effect in our exposed 6‐month‐old groups. The differential expression of *Smad3* suggests possible alterations in cell survival due to its role in cross talk between Notch and TGF‐beta signaling (Kunxin, 2008). We demonstrate that disrupted regulation in the Notch pathway may play a key role in the development of metabolic disease and accelerated aging associated with fetal programming of adult disease.

Nonalcoholic fatty liver disease (NAFLD) can develop as a complication of metabolic syndrome or consequence of aging, and intrauterine exposure to maternal malnutrition may be a predisposing risk factor for NAFLD (Shankar *et al*., 2010; Alisi *et al*., 2011). The primary histological finding is lipid droplet accumulation in hepatocytes, and 20–30% of this lipid deposition is due to *de novo* lipogenesis, a function carried out by the liver parenchyma (Hanson, 1967). The upregulation of the pro‐lipogenic genes, *Prkcb* and *P*c, in the liver of our 6‐month exposed and unexposed aging animals is consistent with reports of hepatocyte‐specific fatty change in NAFLD. Although these are not canonical fatty acid metabolism genes, *Prkcb* and *P*c are involved in lipid homeostasis and regulation (Jitrapakdee, 1999; Huang *et al*., 2012). Increased expression of protein kinase C family proteins, in the liver, is associated with obesity and insulin resistance in mouse and humans (Bezy *et al*., 2011). Furthermore, *Prkcb* depletion in *ob/ob* mice reduced lipid content of liver and muscle and significantly prevented the rise in liver triglyceride levels that was induced by high‐fat feeding (Huang *et al*., 2012). Pyruvate carboxylase (PC) serves as an essential enzyme in lipogenesis because it provides a significant proportion of NADPH, needed for fatty acid synthesis, through its generation of oxaloacetate (Jitrapakdee, 1999). Interestingly, *P*c was found to have fourfold increased expression in the livers of obese Zucker rats (Jitrapakdee *et al*., 1998). We find that this gene is increased nearly twofold in the liver of our 6‐month exposed and unexposed old animals compared to young controls. Our results highlight the potential for reprogramming of *Prkcb* and *P*c methylation levels in the hepatocytes of maternal undernutrition and overnutrition exposed animals at an early age, making them more prone to lipogenesis and thus reinforcing the advanced aging phenotype hypothesis.

Intriguingly, we found differential methylation in *Irs2* and *Foxa*3, both of which are involved in insulin sensitivity (Ma *et al*., 2014). While we see an increase in their expression in unexposed old animals, we do not see this increase in 6‐month‐old exposed animals. This implies that these two genes are not involved in the early stage of disease development or that the 6‐month‐old animals have not yet reached a sufficient level of dysregulation at these sites.

We employed a genomewide approach to identify DNA methylation, in liver, associated with exposure to extremes in maternal nutrition. A drawback from using liver tissue is that there can be variations in cell composition with age and disease development (Schmucker, 1998). Each cell type can have distinct methylation profiles, and therefore, methylation differences can be a result of shifts in cell populations rather than changes in methylation within hepatocytes. While the methylation profiles could be validated at a cell‐specific level using microdissection of hepatocytes from liver tissue, we believe that the concordance with our gene expression changes make it unlikely do be solely due to a shift in cell composition.

This study is unique in its ability to compare the DNA methylation profiles and gene expression levels between unexposed Old animals and two distinct time points following intrauterine exposures of under‐ and overnutrition. By narrowing our candidate genes to those that overlap between one of the two extremes of exposure to that of aging, we were able to identify biologically relevant and novel genes associated with fetal programming. Additionally, these newly identified genes and pathways have not yet been implicated in the aging process itself and should be assessed for their role in aging alone. One of the key findings of this study is our ability to link our methylation markers to functional consequences at a later time point. This is exemplified by the 6‐month under‐ and overnutrition cohorts showing a similar or exaggerated pattern of expression between our exposed and aging animals for our targeted genes. Our findings support both the advanced aging phenotype and cellular memory hypotheses by demonstrating long‐term consequences of early exposure propagated throughout the lifespan mediated by epigenetic modifications.

## Experimental methods

### Established animal models of maternal calorie restriction and maternal high‐fat feeding

This study protocol was reviewed and approved by the Animal Care and Use Committee of the Albert Einstein College of Medicine. We utilized established models of maternal undernutrition (Torrens *et al*., 2014) (pair‐fed 50% of standard chow consumed by controls starting on gestational day 11 and continued through lactation) and maternal high‐fat feeding (overnutrition) (virgin dams fed diet starting at 3 weeks and continued through pregnancy and lactation) known to result in decrease in insulin sensitivity and metabolic abnormities in adulthood (Sen & Simmons, 2010). Undernutrition dams were started on their experimental diet on gestational day 11 to ensure successful conception and pregnancy. Control animals were fed *ad libitum* standard chow (Diet 5001) consisting of 13.5% fat, 58% carbohydrate, and 28.5% protein, respectively. High‐fat chow consisted of total calories derived from 42.1% fat, 42.7% carbohydrate, and 15.2% protein, respectively (Harlan Tekland, TD.88137). Both diets are fully supplemented with vitamins and minerals. All Sprague‐Dawley female rats were bred with Sprague‐Dawley male rats between 12 and 16 weeks of age and were allowed to deliver spontaneously. Each dam continued on their study diet during pregnancy and lactation. Litters were culled to 8 on postpartum day 1. Except during mating and lactation, all animals were housed in individual cages and subjected to standard light (6am to 6 pm) and dark (6p to 6am) cycles. All animals were weaned at 3 weeks of age and placed on standard control diet *ad libitum*. Old animals were aged to ~20 months on standard chow.

### Genomewide DNA methylation assay

HELP‐tagging assay was performed after isolation of genomic DNA from liver of Con, UN, ON, and Old rats and digested to completion by either HpaII or MspI. The digested DNA was ligated to two custom adapters containing Illumina adapter sequences, an EcoP15I recognition site, and the T7 promoter sequence. Using EcoP15I, we isolated sequence tags flanking the sites digested by each enzyme, methylation‐sensitive HpaII or methylation‐insensitive MspI, followed by massively parallel sequencing of the resulting libraries (Illumina Technology)^27^. HpaII profiles were obtained for each sample (*n *=* *24), calculating methylation scores using a previously generated MspI Rattus norvegicus reference.

### Data processing and statistical analysis

DNA methylation scores from 0 (fully methylated) to 100 (unmethylated) were filtered by confidence score. These confidence scores were calculated for each sample based on the total number of HpaII‐generated reads as a function of the total number of MspI‐generated reads, excluding loci for which the confidence score was lower than the expected mean by locus. For one control and one overnutrition sample, there was an overrepresentation of zero values, to prevent skewing, and this sample was removed from the analysis.

Candidate differentially methylated loci were identified using two‐sided *t*‐tests when comparing control to each other group separately to define locus‐specific differences in average methylation between groups, and the following comparisons were made as follows: Con/UN, Con/ON, Con/Old. After confidence score filtering, the number of testable loci decreased from >1.6 million to 617998 (Con/ON), 709778 (Con/UN) 710027 (Con/Old). To determine the appropriate analytical cutoffs, we conducted a power calculation by running 1000 permutations using *n* = 5 in each group and standard deviation of 10%. This simulation confirmed a power of >80% to detect an absolute difference of 30% of methylation change at a significance level of *P* < 0.005. We defined candidate differentially methylated loci to have an absolute difference between mean DNA methylation scores >30% and a *P* value <0.005 (*t*‐test), calculated using ‘rowttest’ function from ‘genefilter’ package using ‘R’ software (2.15.0). Enrichment for hypo‐ or hypermethylated loci was assessed using Fisher's exact test. Thresholds for nonrandom association were estimated using permutation, and briefly, methylation ratios were sampled 400 times generating a distribution of permuted *P‐*values. Next, loci with *P*‐values falling in the top 97.5% of the permuted *P*‐value distribution were considered as significant after correction (two‐tailed *P* < 0.05). The number of permutations was chosen to encompass the possible combinations when distributing 10 samples in two groups with no replacement. All loci associated with change in gene expression were preserved after this correction. Differentially methylated regions were identified using ‘dmrFind’ function within the charm package (Aryee *et al*., 2011). We applied a gap distance of 10000 bp which was decided based on accommodating the average distance of HpaII sites in the rat genome (2000 bp) and the loss of loci coverage between groups.

### Functional enrichment analysis

To perform GSEA (Subramanian *et al*., 2005), we first linked RefSeq genes (rn4) to our candidate differentially methylated loci. Differentially methylated loci overlapping candidate regulatory regions up to 10‐kb upstream of gene transcription start site and within gene bodies until transcription end site were used to link DNA methylation changes with specific genes. We validated a majority of these pathways using the Bioconductor package *GOseq* to control for bias because of the variation of number of HpaII sites associated with different genes. *P‐*values were calculated for over or underrepresentation using the random sampling method (Young *et al*., 2010).

### Quantitative RT–PCR

Total RNA was extracted from liver of 6 rats from each of all 7 groups (described in Animal Model) using TRIzol (Invitrogen). Integrity was confirmed using gel electrophoresis. cDNA was generated using SuperScript III Reverse Transcriptase kit (Invitrogen), and equal amounts of RNA were used in each cDNA reaction. Real‐time quantitative PCR was carried out in triplicate using LightCycler480 SYBR Green Master Mix (Roche) with a LightCycler480 PCR system. Results were normalized to RPS3 expression and expressed as fold change compared with 8‐week‐old control or 6‐month control rats, where specified, using the 2^−ΔΔCt^ method. Table S2 shows mean+/− standard error of the mean (SEM) (primers listed in Table S1). Statistical analysis was completed using ANOVA and Tukey's HSD test.

## Author contributions

J.N.T., H.J.H., and F.D. performed the experiments and analyzed data. Y.M.Z. and L.C. performed the experiments. J.N.T. wrote the manuscript. H.J.H., F.D., N.B., and F.H.E. guided and designed the study and wrote the manuscript.

## Funding

Support for this project was provided by Reproductive Scientist Development Program (NICHD K12HD00849‐26) and March of dimes. Support was also provided by core laboratories of the Einstein Diabetes Research Center (DK020541), the Einstein Nathan Shock Center (P30AG038072), the Einstein's Medical Student Training Program award (NIH NIGMS T32 GM007288), and Einstein's Center for Epigenomics, including the Epigenomics Shared Facility and Computational Epigenomics Group. The funders had no role in study design, data collection and analysis, decision to publish, or preparation of the manuscript.

## Conflict of interest

None declared.

## Supporting information


**Fig. S1** Randomized methylation profiles.
**Fig. S2** Euler diagram of overlapping loci and genes.
**Fig. S3** Representative differentially methylated region.
**Table S1** Quantitative PCR primers.
**Table S2** Relative expression values.
