# Supplementary material for: Advanced aging phenotype is revealed by epigenetic modifications in rat liver after in utero malnutrition
Source: Aging Cell. 2016 Jul 29;15(5):964–72. doi: 10.1111/acel.12505 (PMC5013021; doi:10.1111/acel.12505)
Supplement: Supplementary file 1 — Fig. S1 Randomized methylation profiles. Fig. S2 Euler diagram of overlapping loci and genes. Fig. S3 Representative differentially methylated region. Table S1 Quantitative PCR primers. Table S2 Relative expression values. [file ACEL-15-964-s001.docx]

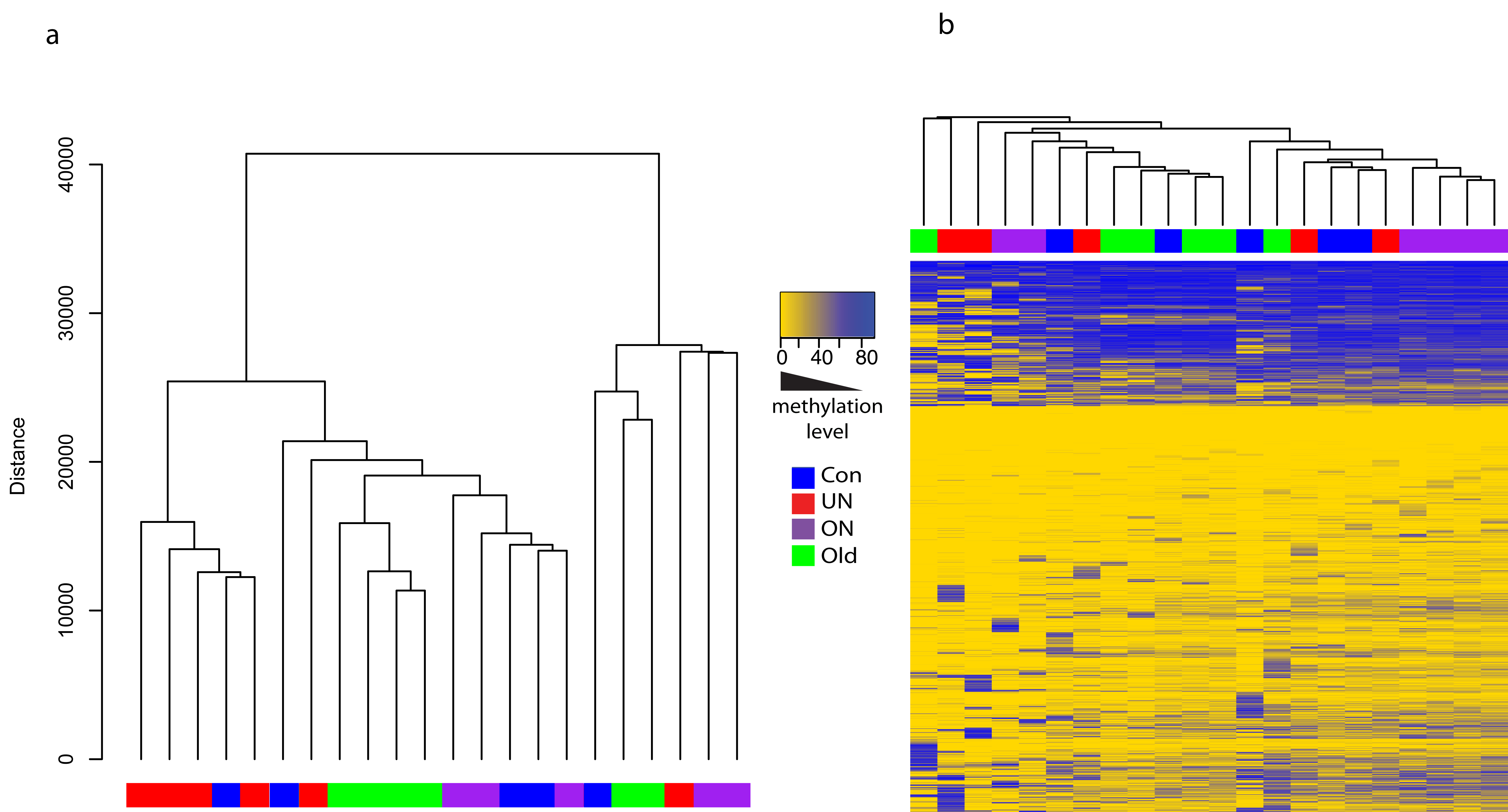


**Supplementary Figure 1.** Dendrogram representing the methylation patterns from all available loci found in each of the four groups (a). Unsupervised clustering analysis of 1906 random loci in each of the four groups (b).


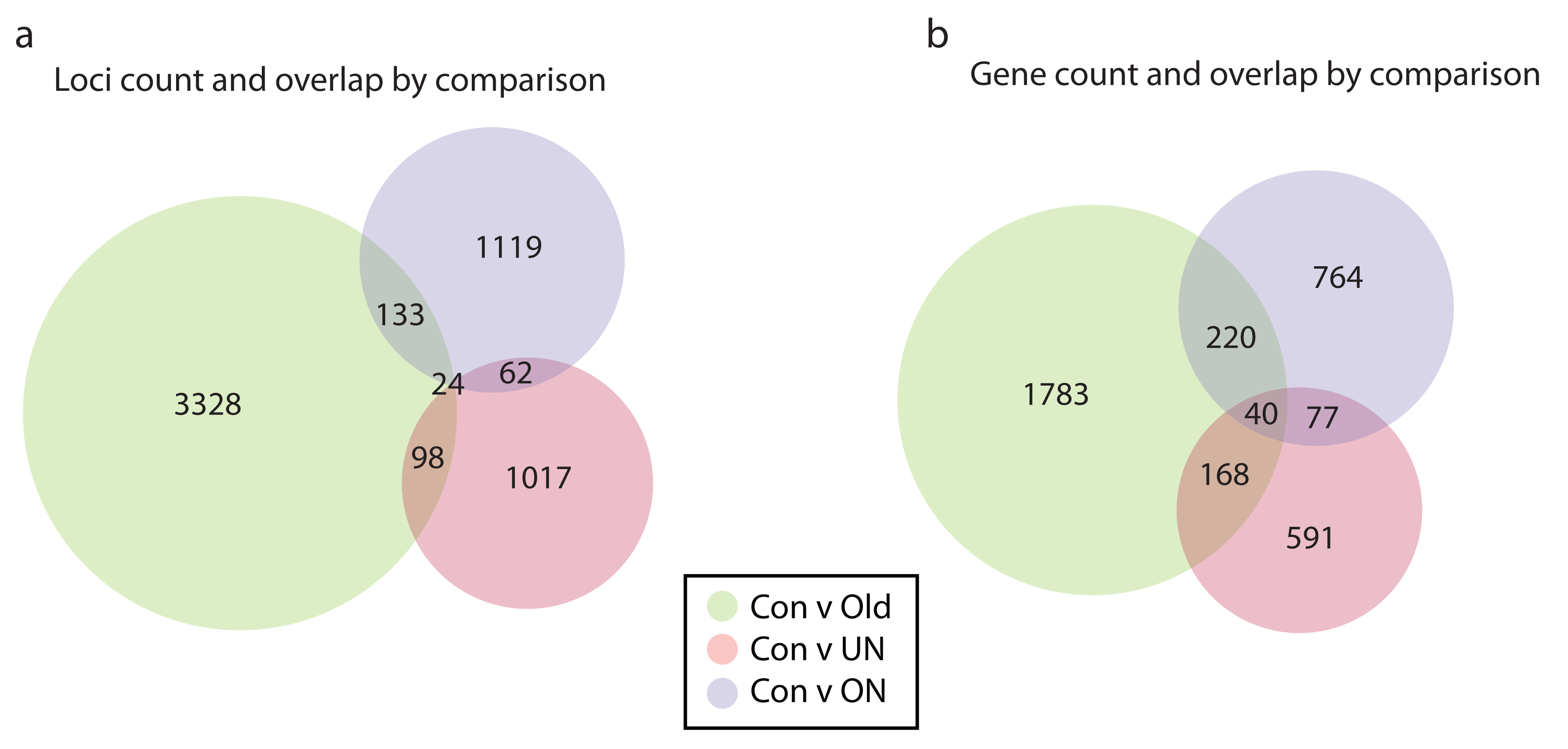


**Supplementary Figure 2.** Euler diagram of significantly differentially methylated loci (a) and genes (b) determined by methylation absolute difference > 30 and significance (p-value < 0.005) thresholds.


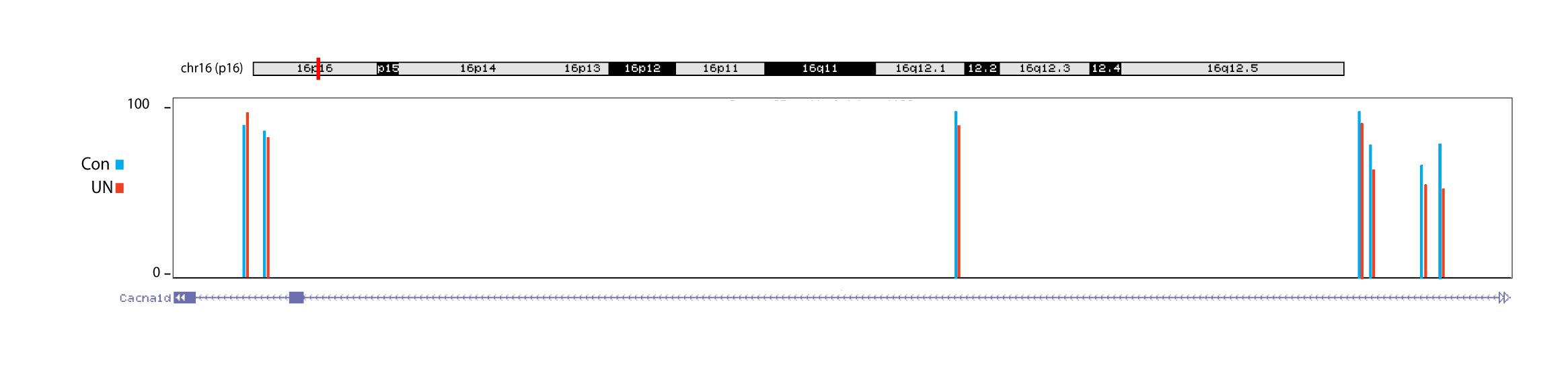


**Supplementary Figure 3.**  Representation of differentially methylated region (DMR) called between Control (Con) and under-nutrition (UN) 9-week rats in *Cacna1d*, this 8.9kb region contains six consecutive CpGs with an average decreased methylation of 12.5% in UN compared to Con.


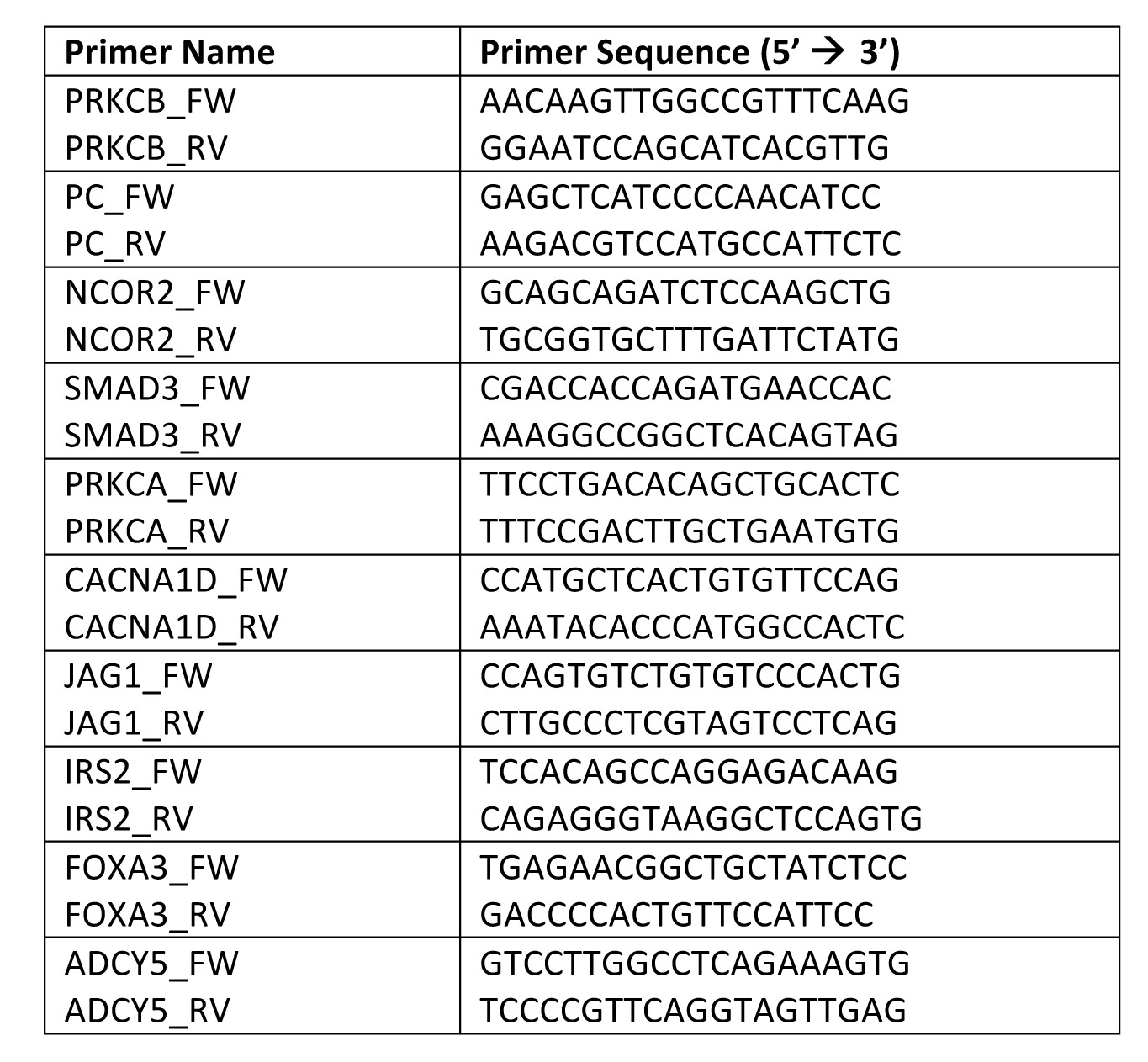


**Supplementary Table 1.** Primer sequences for quantitative PCR.


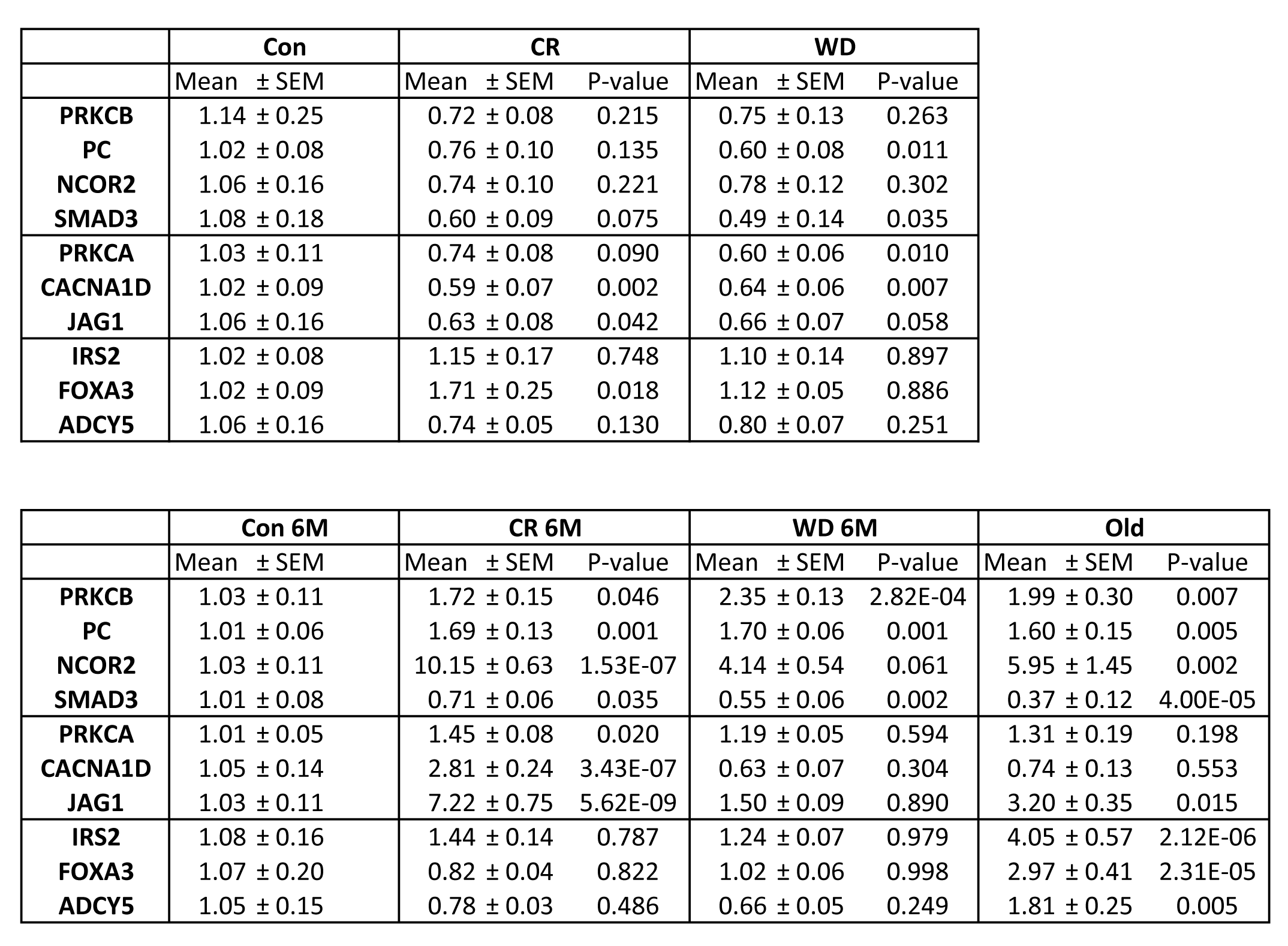


**Supplementary Table 2.** Relative expression of genes with significant differential methylation. Ct values were normalized to RPS3 housekeeping gene. Relative expression was measured in comparison to Control at 9 weeks or 6 months. Mean relative expression with standard error of the mean (SEM). Significance determined by ANOVA.
